# Adding to the Burden: Gastrointestinal Symptoms and Syndromes in Multiple Sclerosis

**DOI:** 10.1155/2013/319201

**Published:** 2013-09-17

**Authors:** David J. Levinthal, Ambreen Rahman, Salman Nusrat, Margie O'Leary, Rock Heyman, Klaus Bielefeldt

**Affiliations:** ^1^Division of Gastroenterology, Hepatology, and Nutrition, Department of Medicine, University of Pittsburgh Medical Center, S848 Scaife Hall, 3550 Terrace Street, Pittsburgh, PA 15261, USA; ^2^Department of Medicine, University of Pittsburgh Medical Center, Pittsburgh, PA 15261, USA; ^3^Division of Gastroenterology, Department of Medicine, University of Oklahoma Health Sciences Center, Oklahoma City, OK 73104, USA; ^4^Neuroimmunology/Multiple Sclerosis Division, Department of Neurology, University of Pittsburgh Medical Center, Pittsburgh, PA 15261, USA

## Abstract

*Background*. Multiple sclerosis (MS) patients often suffer from gastrointestinal (GI) symptoms. However, the full extent and prevalence of such symptoms are not clearly established. Thus, we sought to define the prevalence of GI symptoms and syndromes in those with MS. *Methods*. 218 MS patients completed self-reported demographic and clinical data questionnaires as well as several standardized surveys probing MS severity and GI health. *Results*. Nearly two thirds (65.6%) of patients endorsed at least one persistent GI symptom. Constipation (36.6%), dysphagia (21.1%), and fecal incontinence (15.1%) were common. Surprisingly, nearly 30% (28.4%) of the patients reported dyspeptic symptoms. Using validated diagnostic algorithms, patients met criteria for functional dysphagia (14.7%), functional dyspepsia (16.5%), functional constipation (31.7%), and IBS (19.3%), among others. Functional dysphagia, functional dyspepsia, and IBS were significantly more common in those with self-identified mood disorders. *Conclusions*. Constipation, fecal incontinence, and dysphagia are indeed frequent symptoms seen in MS patients. We also noted a ~30% prevalence of dyspepsia in this population. The mechanisms driving this association are not clear and require further study. However, due to this high prevalence, dyspeptic symptoms should be incorporated into the routine assessment of MS patients and, if found, may warrant collaborative referral with a GI specialist.

## 1. Introduction 

Historically, the routine evaluation of symptoms in MS patients focused on skeletal muscle impairments that restricted mobility. Within the last two decades, symptoms such as dysphagia, bladder and bowel dysfunction, among others, have been increasingly recognized and incorporated into patient assessments [1–3]. The GI problems felt to be most common in MS patients involve deglutition and defecation and require volitional muscle coordination. This association may link the development of such GI problems to underlying MS disease progression. However, GI symptoms that are not dependent upon skeletal muscle control are common in the general population and may also be present in MS patients. More than two decades ago, Hinds and colleagues described a high prevalence of anorectal dysfunction in a large cohort of MS patients [4]. Since then, the diagnostic and clinical approach to MS care has changed dramatically and now emphasizes the early introduction of disease modifying therapies [5]. However, despite changes in MS care, anorectal dysfunction and swallowing problems continue to be an important problem for MS patients [1, 2]. Little is known about other GI symptoms in MS patients in this new era of pervasive use of disease modifying therapies. To address this gap in knowledge, we sought to define the prevalence of GI symptoms and syndromes in a large sample of contemporary MS patients.

## 2. Materials and Methods 

We recruited outpatients from the Division of Neuroimmunology/Multiple Sclerosis, Department of Neurology at the University of Pittsburgh Medical Center between March and October 2012. Consecutive patients were recruited to participate either during routine follow-up clinic visits or during scheduled infusions. All patients eligible for the study carried a clinical diagnosis of MS from their treating MS center neurologist. Patients completed surveys addressing demographic and clinical data, including a focused list of comorbid conditions including gastrointestinal disorders, neurogenic bladder, anxiety, or depression. They also completed validated questionnaires determining the impact of their MS, questionnaires determining the presence of gastrointestinal symptoms and syndromes, and those specifically assessing the severity of fecal incontinence or dysphagia. The protocol was approved by the Institutional Review Board of the University of Pittsburgh.

*Demographic and Clinical Data.* Participants listed their age, sex, ethnic background, marital and employment status, prior education, duration of MS illness, diagnosed subtype of MS, current MS pharmacotherapy, and comorbid medical conditions. 

*The Multiple Sclerosis Impact Scale (MSIS-29)* is a 29-item instrument rating physical (*n* = 20) and psychological (*n* = 9) symptoms on a 5-point Likert scale, with high scores indicating increased impairment; it generates a summary score with physical and psychological subscores [6]. 

*The Adult Functional GI Disorders Rome III Questionnaire* is used extensively in epidemiologic and clinical studies and is composed of 93 questions probing the presence, severity, and duration of various gastrointestinal symptoms [7]. A scoring algorithm applies consensus criteria to diagnose functional gastrointestinal disorders. 

*The MD Anderson Dysphagia Inventory* includes 20 items assessing emotional, functional, and physical problems related to swallowing [8] on a 5-point Likert scale, with high scores indicating better function. 

*The Fecal Incontinence Severity Index (FISI)* is a simple patient assessment tool that calculates severity scores based on descriptions of incontinence frequency specifically for gas, mucus, liquid, and solid materials. Scores are adjusted to reflect their relative impact on quality of life [9]; higher scores reflect worsening incontinence.

### 2.1. Statistical Analysis

Continuous data is presented as mean ± SEM. Group comparisons were performed using SigmaStat 2.0 (SPSS, Chicago); Chi Square, Fisher Exact, and Wilcoxon Rank Sum tests were used as appropriate. Associations were determined using Spearman correlation coefficients. *P* < 0.05 was considered significant for all tests.

## 3. Results

### 3.1. Study Participants

Two-hundred eighteen patients were included in the study (Table 1). Most participants were women (77.9%), Caucasian (89.4%), and married (59.6%). Nearly half were actively employed (48.6%). Patients showed a relatively high level of educational training with only high school diplomas obtained by 48 (22.1%), at least some college experience obtained by 50 (22.9%), a bachelor's degree obtained by 63 (28.9%), and at least some postgraduate education obtained by 56 (25.7%) patients. MS had been diagnosed at an average of 13.3 ± 0.6 years prior to survey completion. The majority described their disease as relapsing-remitting (70.6%). Primary (4.1%) or secondary progressive (11.0%) forms accounted for about 15% of the sample; 32 participants (14.7%) were unsure of disease subtype.

Patients endorsed a variety of comorbid disorders (Table 2). Mood disorders were common with depression (36%) and anxiety (28%) reported by patients; 18% of patients listed both depression and anxiety. The most frequently endorsed gastrointestinal disorders were constipation (40%) and fecal incontinence (7%), with constipation coexisting with fecal incontinence in 4% of participants. Gastroesophageal reflux disease (GERD) was reported by 15% of patients. Thirty-seven (17%) patients had been diagnosed with a neurogenic bladder. 

### 3.2. MS Severity

MSIS-29 responses showed that ~15% of patients reported no physical limitations, and one third of the participants did not experience limitations when ambulating indoors. Consistent with these findings, about 40% did not require help with daily activities. Conversely, more than half (62%) of the patients reported at least moderate problems with strenuous physical tasks, and 40% of the participants experienced moderate or more significant limitations when ambulating. Eighty-three (38%) patients were bothered by relying moderately or greatly on others for daily activities. Given this distribution of MS severity in the sample population, the average MSIS-29 score reflected a moderate impact of the MS on physical and psychological functionings (Table 1). 

### 3.3. Gastrointestinal Symptoms

Our analyses were based upon patient reported symptoms and diagnoses. Thus, we first examined the internal consistency of responses to increase the confidence that these reports were accurate. For example, 83% of the patients endorsing the diagnosis of constipation also responded positively to the presence of constipation for at least 6 months in the Rome III questionnaire, which was significantly higher than in the remaining group (47%; *P* < 0.001). Similarly, 69% of MS patients with fecal incontinence confirmed the presence of accidental leakage of liquid stool within the last 3 months compared to 31% of the rest of the cohort (*P* < 0.01). The MSIS-29 also assesses urgency, with significant differences between groups defined by reported diagnosis of fecal incontinence (2.4 ± 0.1 versus 3.7 ± 0.2; *P* < 0.001). Heartburn frequency rated in the Rome III questionnaire was significantly higher in individuals with known GERD compared to the rest of the study group (3.1 ± 0.4 versus 0.8 ± 0.1; *P* < 0.001). The presence of dysphagia, as rated by the Rome III questionnaire, was correlated significantly with physical domains of the MD Anderson Dysphagia Inventory such as cough with swallowing (*R* = −0.35), time required to complete a meal (*R* = −0.44), and effort swallowing (*R* = −0.36) all showing expected inverse relationships. Lastly, we divided the study cohort based on self-reported mood disorders and compared ratings on subscales of the MSIS-29. Participants with preexisting anxiety had lower ratings of quality of life related to feeling tense or anxious (2.2 ± 0.1 versus 3.2 ± 0.2; *P* < 0.001). The diagnosis of depression was similarly associated with lower quality of life ratings related to feeling depressed during the last 2 weeks (1.8 ± 0.1 versus 2.9 ± 0.2; *P* < 0.001). Consistent with these data, the MSIS-29 psychological subscore was significantly higher (i.e., worse) in patients with a reported mood disorder (Table 2). Therefore, the patient reports used in this study appear to be internally consistent across multiple symptom domains of the standardized questionnaires. 

**Figure 1:**
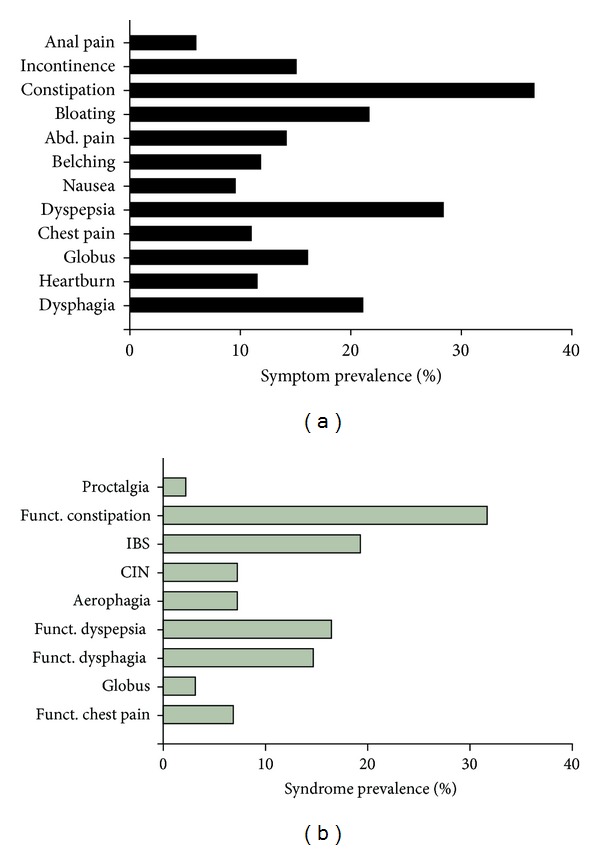
Prevalence of gastrointestinal symptoms (a) or defined syndromes based on Rome III criteria (b) in MS patients (*n* = 218). Only symptoms and syndromes with a prevalence of at least 3% were included in the graph. CIN: chronic idiopathic nausea; IBS: irritable bowel syndrome.

Using the validated Rome III questionnaire to assess the prevalence of GI symptoms in our cohort of MS patients, we found that 66% of participants endorsed the presence of at least one GI symptom. Constipation and fecal incontinence were among the most commonly reported symptoms, reported by 37% and 15% of patients, respectively, (Figure 1(a)). Additionally, 46 MS patients (21%) experienced at least monthly episodes of dysphagia, and 47 patients (22%) rated coughing spells in conjunction with swallowing as moderately severe or worse based on responses to the MD Anderson Dysphagia Inventory. Twenty-five (12%) patients experienced at least weekly heartburn, with 12 of these participants also reporting swallowing problems. Frequent odynophagia was less common (*n* = 7, 3%) and was associated with heartburn in all but one patient. At least weekly episodes of globus sensation were endorsed by 35 (16%) participants. 

Notably, 62 (28%) of the participants reported at least one dyspeptic symptom. Dyspepsia includes symptoms of early satiation, postprandial fullness, and epigastric pain or burning. In our patient cohort, the most common specific dyspeptic complaints were early satiation (17%) and postprandial fullness (16%), with 17 patients (8%) reporting both. Additionally, patients reported at least weekly episodes of epigastric pain (9%). Nausea was reported by 10%, while vomiting was less common (2%). Frequent and bothersome belching affected 12% of the participants at least weekly. Bloating occurred weekly or more often in 22% of the patients. Lastly, at least weekly episodes of pain located in the chest (11%), in the abdominal area (14%), and in the anal area (6%) were other relatively common problems.

### 3.4. Functional Gastrointestinal Syndromes

Using symptom clusters, we determined the prevalence of functional gastrointestinal disorders in the MS patient sample based on the currently accepted diagnostic algorithms [7] (Figure 1(b)). Fifteen patients (7%; men = 0) met criteria for functional chest pain of presumed esophageal origin. While none of these participants endorsed the presence of heartburn, several listed GERD as a comorbid condition. As we did not have access to endoscopic or other diagnostic tests, we could not truly differentiate functional chest pain from functional heartburn or gastroesophageal reflux disease. Globus was present in only 7 (3%; men = 1) patients. The high prevalence of deglutitive abnormalities in MS patients confounds the assessment of functional dysphagia, which by definition focuses on esophageal rather than oropharyngeal symptoms. However, given this caveat, 32 (15%; men = 10) patients met criteria for functional dysphagia. Functional dyspepsia was present in 36 (17%; men = 10) patients. Nine patients (4%, men = 2) met criteria for postprandial distress syndrome, and none for epigastric pain syndrome. Unspecified excessive belching or aerophagia was present in 16 (7%; men = 0), chronic idiopathic nausea in 16 (7%; men = 2), chronic idiopathic vomiting in only 3 (1%; men = 0), and cyclic vomiting syndrome in 5 (2%; men = 0) patients. A total of 42 (19%; men = 11) patients met criteria for IBS, and an additional 5 (2%) patients had functional diarrhea. Functional constipation was present in 69 (32%; men = 14) and fecal incontinence in 33 (15%; men = 4) participants. Two (1%) patients met diagnostic criteria for functional abdominal pain and an additional 5 (2%) patients for functional anal pain. None of the participants fulfilled criteria for biliary pain syndromes.

### 3.5. Correlations and Subgroup Analyses

**Figure 2:**
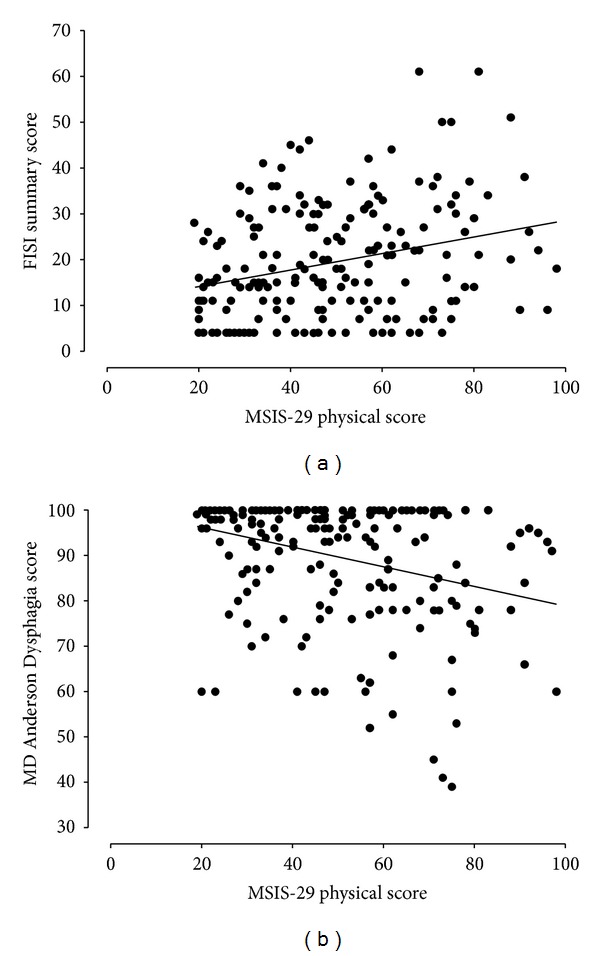
Association between the physical score of the MSIS-29 and scores on the Fecal Incontinence Severity Index (FISI) (a) (*r* ^2^ = 0.28, *P* < 0.01) or the MD Anderson Dysphagia Inventory (b) (*r* ^2^ = −0.36, *P* < 0.01).

A previous study reported increased rates of gastrointestinal dysfunction as a function of MS disease duration and severity [4]. Thus, we investigated this relationship in our MS cohort. Disease duration did not significantly correlate with the physical subscore of the MSIS-29 (*R* = 0.06) nor the presence or severity of GI symptoms (data not shown). The physical MSIS-29 subscore did not correlate significantly with the presence of fecal incontinence (*r* ^2^ = 0.04) or dysphagia (*r* ^2^ = 0.03) as determined by the Rome III questionnaire. However, more detailed assessments of symptom severity did show a statistically significant relationship between severity ratings and the global impairment of physical function determined by the MSIS-29 subscore (Figure 2). Prior studies determined an increasing prevalence of anorectal dysfunction in MS patients with bladder dysfunction [4]. Thus, we examined the prevalence of constipation and/or fecal incontinence in patients reporting a diagnosis of neurogenic bladder (*n* = 37). Interestingly, the reported prevalence of defecation problems (constipation—*n* = 16 (43%); fecal incontinence—*n* = 7 (19%)) was not higher in this subgroup compared to the rest of the cohort. The severity rating of nausea was correlated with the MSIS-29 summary score (*r* = 0.31, *P* < 0.01) and the physical (*r* = 0.25; *P* < 0.01) and mental subscores (*r* = −0.37; *P* < 0.01). Similarly, postprandial fullness showed significant correlation with the MSIS-29 scores (summary score: *r* = 0.31; physical score: 0.25; mental score 0.36; *P* < 0.01 for all correlations), while epigastric pain did not correlate significantly with any of the MSIS-29 scores. 

Disease-modifying agents and other medications have potential adverse effects that confound the attribution of gastrointestinal symptoms to MS disease itself. The majority of the cohort was receiving natalizumab (*n* = 163) and thus did not allow a meaningful comparison of symptoms with smaller number of patients using glatiramer (*n* = 21) or interferon therapy (*n* = 16). Smaller numbers of patients were using modafinil and the potassium channel blocker dalfampridine, both of which have been associated with a high likelihood of gastrointestinal symptoms [10, 11].

Considering the high prevalence of anxiety and/or depression in patients with functional gastrointestinal disorders [12–14], we examined the correlation between functional gastrointestinal syndromes and reported diagnoses of a mood disorders. The symptom clusters characterizing functional dysphagia, functional dyspepsia, and IBS were all statistically (i.e., *P* < 0.05) more common in MS patients who reported a comorbid mood disorder (Table 3).

## 4. Discussion

Gastrointestinal problems linked with underlying musculoskeletal dysfunction, such as dysphagia and defecation disorders, are common in patients with inherited and acquired neurologic disorders [15–20]. Our data lends further support to this association, as our findings corroborate previous data demonstrating a high prevalence of dysphagia and defecation disorders in the MS population [3, 4, 21, 22]. However, by using a comprehensive approach using validated survey tools, we now provide new and important insight demonstrating that MS patients experience a more extensive burden of GI symptoms than previously recognized. For example, we identified a high prevalence of dyspeptic symptoms and pain that far exceed rates reported in the general population [23–25].

About 20% of our cohort experienced dysphagia, consistent with prior reports [26, 27]. Our approach cannot reliably distinguish oropharyngeal dysphagia from esophageal dysphagia, but our findings suggest that impaired deglutition is the underlying problem in the majority of our study participants. While oropharyngeal dysphagia is known to be more common in individuals with neurologic disorders in general, we base this interpretation on several objective findings. First, coughing spells with swallowing were endorsed by nearly all patients concurrently reporting dysphagia. Second, only about one-third of the MS patients with dysphagia complained of esophageal symptoms such as heartburn or odynophagia. Lastly, reflux symptoms or a diagnosis of GERD, the most common cause of esophageal dysphagia, were reported by MS patients at rates similar to those seen in the general population [28].

Bowel dysfunction is another common problem in MS patients, and symptoms of anorectal disorders have been incorporated into routine clinical assessment tools [1]. Constipation and/or fecal incontinence were present in about ~50% of the MS patients examined, with symptom severity correlating with the overall impact of MS on physical function. The high prevalence and relationship with disease progression fit into the context of previously published results [4, 29–35] and exceeds reported rates of constipation and fecal incontinence in the general population [36, 37]. Considering differences in recruitment strategies, questionnaires, and definitions of primary endpoints in prior studies in the MS population, we cannot directly compare our contemporary results in order to determine time trends that may reflect a potential impact of disease modifying therapy. 

Our study extended previous investigations to examine the prevalence of multiple different symptoms related to GI problems. Using a validated survey, we found a high prevalence of dyspeptic symptoms in MS patients approaching 30% a rate that far exceeds the ~8% prevalence of dyspepsia found in the general population [38]. Such dyspeptic symptoms have previously been reported in small case series of patients with MS or related illnesses. For example, severe vomiting has previously been described in patients with neuromyelitis optica and demyelinating lesions in the area postrema [39], and there are case reports of MS patients with comorbid gastroparesis [40, 41]. In addition to such potential underlying mechanisms, MS patients often receive disease modifying and complex symptom-oriented medical therapy, which may contribute to the development of dyspeptic symptoms [10, 11, 42, 43]. Such a mechanism is supported by the higher ratings for nausea in patients in our cohort in the few patients who listed modafinil or dalfampridine as part of their treatment. As our study design did not include diagnostic testing or review of patient charts, the drug use listed by study participants may be incomplete. Despite these shortcomings, our data provide the first estimate of the prevalence of dyspeptic symptoms in a large cohort of MS patients, who were not preselected for the presence of GI problems. 

Furthermore, chest, abdominal, or anal pain was also relatively common in our cohort. While many studies have described the common occurrence of pain in MS in 30–50% of patients, the location is typically reported in the extremities, head, or back and is considered neuropathic in the majority of cases [44]. In our cohort, abdominal pain was associated with other GI symptoms and may thus represent some of the defined functional GI syndromes such as functional dyspepsia or irritable bowel syndrome. While such functional illnesses are defined by symptom clusters, as assessed in our study, the existence of an underlying neurological illness raises the question whether specific disease mechanisms may differ in this patient group. First, these symptoms may be manifestations of the MS illness itself, perhaps related to impaired neural activity in brain or spinal pathways involved in autonomic function or sensation. Alternatively, patients may experience dyspeptic symptoms as a consequence of medications used to treat MS or other comorbid conditions, as already discussed above. Lastly, the symptoms or symptom clusters may be related to comorbid conditions, most importantly depression and anxiety. These affective spectrum disorders are commonly found in patients with functional GI diseases and pain syndromes and are likely to play an important role in the manifestations of these disorders via multiple mechanisms including increased somatization, hypervigilance, and catastrophizing [38, 45–48]. Prior studies have determined a high prevalence of mood disorders in the MS population, with rates similar to those of self-reported depression or anxiety in our cohort [49, 50]. Consistent with findings in patients without coexisting neurological problems, the presence of a mood disorder was associated with a higher prevalence of gastrointestinal symptoms in our cohort. Despite the emerging parallels with the general population, more detailed mechanistic investigations are needed to determine causes for these symptoms and syndromes on MS patients.

We recruited a large cohort of patients receiving treatment for MS in a tertiary referral center, which could bias the study population with a disproportionate number of severely affected patients. However, about 50% of the participants were actively employed and the MSIS-29 scores we observed correspond favorably with prior reports [51, 52]. In addition, the disease-specific quality of life, as measured with the MSIS-29, was comparable to several recently published outpatient cohorts or web-based registries [53–56]. The majority of our study patients were receiving infusion therapy (>80%), which likely biased the patient population toward those with a relapsing-remitting subtype. Thus, MS patients with primary progressive disease were likely underrepresented in our sample. However, given that relapsing-remitting disease is the most common MS subtype, our results still are representative of most MS patients. We also have relied exclusively on self-reports, thereby introducing possible, but unavoidable, recall bias or data inaccuracies. However, the Rome questionnaires were specifically designed to perform prevalence studies in different cohorts [57, 58] and include a section on alarm symptoms that suggest the presence of nonfunctional GI disorders. These alarm symptoms were endorsed by fewer than 10% of our total sample (data not shown). Thus, while we did not have access to patient data and cannot exclude structural GI diseases, it is unlikely that such structural diseases account for the high prevalence of GI symptoms reported in our cohort.

Overall, our data have clearly demonstrated that multiple GI symptoms are common in MS patients. These symptoms are not restricted to deglutition and defecation and include frequent dyspeptic symptoms and pain. Our findings have important implications for the management of MS patients, as GI problems significantly impair quality of life and may interfere with MS treatment. We have also uncovered an association between mood disorders and some of the GI problems observed in MS patients. Whether cause or consequence, this particular association not only demonstrates a need for further study, but also points at potential diagnostic approaches and therapeutic targets. Given the prevalence of GI symptoms in MS patients, screening for such symptoms should be incorporated into the routine assessment of these patients, and positive findings may warrant collaborative referral to a GI specialist. 

## Figures and Tables

**Table 1:** Demographic and clinical characteristics of the study MS population.

	All	Women	Men
*N*	218	170	48
Age	47.6 ± 1.0	47.6 ± 1.1	47.2 ± 3.2
Duration	13.3 ± 0.6	13.5 ± 0.7	12.3 ± 1.2
MSIS-29—overall	70.7 ± 1.8 (range 20–140)	70.3 ± 2.0	72.1 ± 4.0
Physical	48.8 ± 1.4	48.3 ± 1.5	50.4 ± 3.0
Psychological	23.0 ± 0.6	23.7 ± 0.8	22.9 ± 4.0
FISI summary	19.1 ± 0.9 (range 4–60)	19.4 ± 0.9	18.0 ± 1.8
Anderson dysphagia	89.8 ± 0.9 (range 39–100)	89.6 ± 1.1	90.3 ± 1.9

GI Symptoms	*N* (%)	*N* (%)	*N* (%)

None	75 (34.4)	54 (31.8)	21 (43.8)
1	67 (30.7)	57 (33.5)	10 (20.8)
2	33 (15.1)	27 (15.9)	6 (12.5)
3	17 (7.8)	10 (5.9)	7 (14.5)
4	14 (6.4)	11 (6.5)	3 (6.3)
>4	12 (5.5)	11 (6.5)	1 (2.1)

**Table 2:** Prevalence of self-reported comorbidities in the study cohort.

Diagnosis	Prevalence
Constipation	88 (40%)
Fecal incontinence	16 (7%)
Gastroesophageal reflux	32 (15%)
Irritable bowel syndrome	11 (5%)
Peptic ulcer disease	4 (2%)
Celiac disease	2 (1%)
Functional dysphagia	1 (0.5%)
Neurogenic bladder	37 (17%)
Depression	78 (36%)
Anxiety	60 (28%)

**Table 3:** Impact of reported affective spectrum disorder on MSIS score, gastrointestinal symptoms, and gastrointestinal syndromes.

	Affective spectrum disorder	No affective spectrum disorder	Significance
*N*	95 (men = 16)	123 (men = 32)	Sex *P* = 0.15
MSIS—overall	76.9 ± 2.8	66.0 ± 2.3	***P < *0.01**
Physical	51.3 ± 2.1	46.8 ± 1.8	*P* = 0.08
Psychological	26.8 ± 1.0	20.2 ± 0.7	***P < *0.01**
Globus	2	5	*P* = 0.67
Chest pain	5	5	*P* = 0.92
Heartburn	10	14	*P* = 0.98
Dysphagia	21	12	***P = *0.02**
Dyspepsia	23	13	***P = *0.012**
Nausea	11	5	*P* = 0.065
Vomiting	2	1	N/D
Belching	9	7	*P* = 0.56
IBS	26	16	***P = *0.013**
Constipation	38	42	P = 0.45
Incontinence	14	19	P = 0.96
